# Organ donation after euthanasia starting at home in a patient with multiple system atrophy

**DOI:** 10.1186/s12910-021-00686-4

**Published:** 2021-09-06

**Authors:** Najat Tajaâte, Nathalie van Dijk, Elien Pragt, David Shaw, A. Kempener-Deguelle, Wim de Jongh, Jan Bollen, Walther van Mook

**Affiliations:** 1 Department of Anesthesiology and Intensive Care Medicine, Zuyderland Medisch Centrum, Heerlen, Netherlands; 2 Department of Intensive Care Medicine, Maastricht University Medical Center, Maastricht, Netherlands; 3 Care and Public Health Research Institute, Maastricht University, Maastricht, Netherlands; 4 Institute of Biomedical Ethics, University of Basel, Basel, Switzerland; 5 Department of Organ Donation Coordination, Maastricht University Medical Center, Maastricht, Netherlands; 6 Department of Anesthesiology, Pain and Palliative Medicine, Radboud University Medical Center, PO Box 9101, 6500 HB Nijmegen, Netherlands; 7 Maastricht UMC+ Academy, Maastricht, Netherlands; 8 School of Health Professions Education, Maastricht Universityr, Maastricht, Netherlands

**Keywords:** Organ donation, Euthanasia, Legal, Transplantation, Case report

## Abstract

**Background:**

A patient who fulfils the due diligence requirements for euthanasia, and is medically suitable, is able to donate his organs after euthanasia in Belgium, the Netherlands and Canada. Since 2012, more than 70 patients have undergone this combined procedure in the Netherlands. Even though all patients who undergo euthanasia are suffering hopelessly and unbearably, some of these patients are nevertheless willing to help others in need of an organ.

Organ donation after euthanasia is a so-called donation after circulatory death (DCD), Maastricht category III procedure, which takes place following cardiac arrest, comparable to donation after withdrawal of life sustaining therapy in critically ill patients. To minimize the period of organ ischemia, the patient is transported to the operating room immediately after the legally mandated no-touch period of 5 min following circulatory arrest. This means that the organ donation procedure following euthanasia must take place in the hospital, which appears to be insurmountable to many patients who are willing to donate, since they already spent a lot of time in the hospital.

**Case presentation:**

This article describes the procedure of organ donation after euthanasia starting at home (ODAEH) following anesthesia in a former health care professional suffering from multiple system atrophy. This case is unique for at least two reasons. He spent his last conscious hours surrounded by his family at home, after which he underwent general anaesthesia and was intubated, before being transported to the hospital for euthanasia and organ donation. In addition, the patient explicitly requested the euthanasia to be performed in the preparation room, next to the operating room, in order to limit the period of organ ischemia due to transport time from the intensive care unit to the operating room. The medical, legal and ethical considerations related to this illustrative case are subsequently discussed.

**Conclusions:**

Organ donation after euthanasia is a pure act of altruism. This combined procedure can also be performed after the patient has been anesthetized at home and during transportation to the hospital.

## Background

Organ donation after euthanasia is legally permitted in the Netherlands since the introduction of the Euthanasia Act in 2002. By December 2020, this combined procedure had been performed more than 70 times. If a patient requests this combined procedure, he needs to undergo several preparatory investigations. Euthanasia is then performed in the hospital by the performing physician—most often his general practitioner—after which the patient’s organs will be procured.

Research demonstrated that an estimated 10% of all patients who undergo euthanasia might be medically suitable organ donors [1]. A more recent comparative study concluded that organs transplanted following euthanasia have superior immediate graft function compared to other DCD-III transplants [2, 3].

The authors present a unique case of organ donation after euthanasia starting at home (ODAEH): a patient who requested to be sedated at home, and to die in the operating room (OR), in an attempt to minimize the warm ischemic time of the organs [4]. This request imposed medical, legal and ethical challenges for the health care professionals involved, which will be consecutively discussed.

## Case presentation

A 63-year old man had been suffering from multiple system atrophy (MSA) for four years, due to which he was physically deteriorating. This led him to request euthanasia. The man was a former health care professional and his last wish was to donate his organs following euthanasia. He was aware of factors influencing the quality of donated organs, like the warm ischemic time (WIT). Consequently, he specifically requested euthanasia to be performed in the operating room.

However, he objected to spending his last conscious moments in a hospital. His explicit wish was to be sedated at home, followed by transportation to the hospital. This way, he could ‘fall asleep’ in his familiar environment, surrounded by his loved ones.

Before the procedure, the patient’s case was discussed several times with all stakeholders, including his general practitioner, the organ donation coordinator (ODC), the hospital’s legal officer, medical director, coordinating intensivist for donation affairs, municipal coroner, public prosecutor, anaesthesiologist-intensivists, the medical ethical committee and the hospital board. All were in favour of facilitating the patient’s last wish.

Anticipatory preparations were necessary to perform general anesthesia at home. The ODC visited the patient and his family multiple times, during which he developed and maintained a close, yet professional relationship with both the patient and his relatives. He discussed the patient’s wishes, provided information about the combined euthanasia and organ donation procedure, and anticipated any practical issues that might arise, including measuring the elevator for the ambulance stretcher and the working space around the patient’s bed. This was especially important because the patient’s family, his wife, and (grand)children wanted to be present during induction of anesthesia. All potential scenarios were discussed with the patient and his relatives to achieve clarity on mutual expectations and to prevent any potential unpleasant or unforeseen experiences for the family. All visits were pre- and post-discussed with the patient’s own general practitioner and all other stakeholders.

On the day of the procedure the team (ODC, anesthetist-intensivists, intensive care nurse) was professionally equipped to provide anesthesia, and perform endotracheal intubation and subsequent mechanical ventilation at the patient’s home. The patient was sitting in his lounge chair in the living room facing a beautiful outdoor environment, with his wife, children, grandchildren and his dog surrounding him, while music was playing in the background.

The patient confirmed his last wish to undergo euthanasia followed by organ donation. After transferring the patient to his bed, he was given an intravenous access. He said farewell to his family, and subsequently the general anesthesia was induced with 15 mg midazolam, 10 mg piritramide and 100 mg rocuronium. Following uncomplicated intubation and mechanical ventilation, he was transported to the university hospital by ambulance (mobile intensive care unit). The patients’ relatives chose to stay at home and not to be present during euthanasia itself.

After the patient’s arrival in the OR’s preparation room, the remaining preparatory investigations were performed, including an abdominal ultrasound. His general practitioner administered the euthanasia drugs according to the euthanasia guideline after which the mechanical ventilation was switched off. After circulatory arrest and the mandated 5-min no-touch waiting period, the patient was declared dead and transported to the operating room, where the surgical retrieval team was anticipating his arrival. Any contact between those performing anesthesia and euthanasia and the surgical team was avoided. Organ procurement was uneventful; both kidneys, the liver and pancreas were donated. The lungs were rejected because of relapsing aspiration pneumonias in the past.

As euthanasia, from a legal perspective, is a non-natural death, the municipal coroner and public prosecutor were asked to grant a priori permission for the combined euthanasia and donation procedure. After the procurement, the patient’s body was brought home again by the mortician. The euthanasia review committee evaluated the procedure post hoc and determined that all due diligence criteria criteria had been fulfilled. All extra costs of the organ donation procedure were reimbursed by the Dutch transplantation society. The costs for the (exceptional) mobile intensive care transport were not charged. None of these measures had consequences for the regular care of other patients.

## Organizational/logistical challenges

The national guideline on organ donation and euthanasia was used as the basis to prepare for organisation of the procedure [5]. The main practical challenge was converging those directly involved in the procedure to the right place at the right time. This is not a procedure that is arranged overnight, and the patient’s preferences regarding its timing were paramount.

Furthermore, the availability of the operating theatre is another influential factor to consider. Once it proved feasible to perform the procedure on the date preferred by the patient, the general practitioner had to sign a hospital admission agreement. The hospital pharmacist prepared the euthanasia drugs prior to the patient’s arrival. A more detailed description of the organisational issues surrounding organ donation and euthanasia has been published previously [6].

## Discussion and conclusions

### Medical challenges

Over 80% of patients undergoing euthanasia choose to die at home [7]. Consequently, the vast majority *will not* opt for euthanasia followed by organ donation as that normally requires dying in the hospital. The patient therefore requested to be sedated at home and to remain anesthetized during transport until the euthanasia was performed in the hospital.

‘Simply’ sedating a patient (without subsequent intubation), using for example low doses of propofol or midazolam, could potentially have negative consequences for the airway and/or breathing resulting in desaturation with possible negative impact on the quality of the donated organs. One decided to intubate the patient, even though intubation is not risk-free either. Intubation carries—among other things—the risk of a failed intubation, loss of a protected airway, aspiration and/or subsequent hypoxia due to ventilation problems. All periprocedural risks were pre- discussed with the patient and his family. In case of periprocedural cardiac arrest it was decided to refrain from resuscitation, and the combined procedure. To lower the risk of aspiration potentially leading to hypoxia and subsequent damage to other organs, the patient was advised not to eat or drink prior to the procedure (analogous to an elective surgical procedure).

Once in the hospital, the euthanasia drugs were administered by his general practitioner—resulting in the death of the patient. In other organ donation after euthanasia procedures, this is often done in the intensive care unit, as this ward is closest to the operating room and the personnel is experienced in organ donation procedures. The five minute *no touch period* ensures that circulation remains absent, respects the dead donor rule, and results in a clear and strict separation between the euthanasia procedure and organ donation procedure.

ODAEH has been described before, but in addition to the home sedation, our patient explicitly requested the euthanasia to be performed in the preparation room next to the operating room to preserve the best quality of his organs [8]. After administration of the euthanasia drugs according to protocol by his general practitioner the patient died within a few minutes.

### Legal challenges

Because it is formally still a punishable offence under the Dutch Penal Code, euthanasia is only permissible if the relevant due diligence requirements are met, as laid down in the Euthanasia Act. These criteria include a voluntary and well-considered request from the patient, unbearable suffering without any prospect of improvement, and the lack of a reasonable alternative [9].

When a patient is anesthetized, one might argue that the requirement of hopeless and unbearable suffering is not applicable anymore. The patient would then in theory, from the perspective of the *letter of the* law, no longer fulfil the due diligence requirements because of which it would become legally impossible to perform euthanasia. However, in terms of the *spirit of the law*, one can reason that the sustained unbearable and hopeless suffering is still present, since it would reappear as soon as the temporary sedation effects wear out. The regional review committees on euthanasia determine *ex post* whether all due diligence requirements were fulfilled in each individual case. In previous ODAEH cases, the review committees judged that all due diligence requirements had been met [7].

If a patient would be anesthetized and transported to the operating room, one could, also merely theoretically, consider removing the organs while the patient is still anesthetized. In our prior experiences, some patients, who request euthanasia and organ donation actively voice such requests, including our patient. Since the patient would not yet be legally dead at the time of organ harvesting, this would imply living organ donation. This type of organ donation has previously been described as ‘organ donation euthanasia’ (ODE), which is currently still illegal, and would result in prosecution [10, 11]. This thus contrasts with euthanasia which is permitted after strict adherence to the due diligence criteria. Not adhering to the latter likewise results in prosecution and punishment. Furthermore, an ODE procedure would infringe the dead donor rule, which states that vital organs should only be taken from persons who are dead. Our patient however objected to visit the hospital for any preparatory investigations that would be necessary to donate the heart, for the same reasons he requested home sedation. Recently, it became possible in the Netherlands to donate the heart during a DCD procedure. This was not possible at the time of death of our patient. In our case additional DCD heart donation, although now legally and medically possible, would furthermore not have been an option since our patient was considered too old to donate his heart. The currently used maximum age for DCD heart donation in the Netherlands is 58 years. Living organ donation of one kidney, followed by a euthanasia procedure is an alternative to ODE, but has, to the best of our knowledge, not been performed in the Netherlands so far.

### Ethical challenges

Many ethicists use four basic principles to assess ethical issues: the principles of *respect for autonomy*, *beneficence*, *non-maleficence*, and *justice*. These are concisely discussed below. A more extensive discussion is beyond the scope of this article, and previously published elsewhere [9].

## Respect for autonomy

The principle of respect for autonomy reflects that the patient must make his or her own decisions independently and according to his or her personal values and beliefs. By respecting the autonomy of our patient, he was able to coordinate his last hours. Facilitating organ donation after euthanasia maximally respects the patient’s autonomy. ODAEH is an even higher level of respecting the patient’s autonomy, and contributed to his request to help other patients in need. Even though several additional medical, legal and logistical challenges are faced, the authors consider it appropriate to at least consider making this extra effort. The current guideline does not provide a formal position and there is not yet a cost benefit analysis for this particular situation.

To respect the patients’ wish to keep the warm ischemic time as short as possible we proceeded with the euthanasia procedure in the preparation room. One potential ethical objection to euthanasia in general, organ donation after euthanasia in particular and specifically this type of case concerns the principle of respect for autonomy. In order to respect autonomy, any consent—whether to donation or euthanasia—must be freely given, without any pressure or coercion. This leads to the concern that patients who otherwise might not choose euthanasia could do so partly because they feel an obligation to stop being a burden on relatives (a general concern), to donate their organs to help others perceived as being in greater need (a general organ donation euthanasia concern), or to donate via home sedation in order to maximise the number and viability of organs being donated (a specific donation concern).

The general concern about the ‘burden’ argument has been dealt with extensively in the literature and we do not address it here as it does not concern organ donation euthanasia but rather the practice of euthanasia in general. The general donation concern is easily dealt with due to the clear separation of the euthanasia consent process from the organ donation consent process; only once the wish to access euthanasia is assessed and approved and consent obtained will there be any discussion of organ donation, which will be addressed by a separate team. The more specific concern about pressure to choose home sedation in order to maximise donation potential is even less plausible. Most patients would not be aware that this is even an option, and it is not normally suggested. Even if a patient were informed about this option, it would be made clear that he or she had no obligation whatsoever to choose this option—just as there is no obligation to consent to donation at all, or to go ahead with euthanasia.

### Beneficence

The ethical principle of beneficence requires the health care providers to act in the best interest of their patients in each situation. To ensure beneficence for our patient we had to consider his individual circumstances, and not just listen to them, but also value his last wishes. Even though his request was extraordinary and so far, not included in the Dutch guideline on organ donation after euthanasia, we decided to honour his request after careful and timely deliberation. From a utilitarian perspective, the decision to honour the last wishes of the patient produced the greatest good for many other patients.

### Non-maleficence

Non-maleficence, means ‘avoiding and/or preventing harm’. The difficulty is in defining the nature of harm, since many types of harm ranging from physical and emotional injury to deprivation of property or violation of rights exist. In health care, harm relates to a more focussed definition including pain, disability or death. Granting the euthanasia request has limited the period of suffering, and thus harm. In the presented case a broader definition of harm is required in the ethical consideration to fulfil the last wishes of the patient. Both the euthanasia and organ donation were explicitly requested by the patient.

### Justice

The principle of justice requires that patients are treated fairly, equitably and with respect. All the health care providers involved in the procedure carried out their tasks in protected time, so conflict with other patients’ care was avoided. Another aspect to consider in the Netherlands is that the patient has no influence on the allocation of his donated organs. Comparable to donation after death, the organs are allocated by Eurotransplant. Since additional human resources and time are required to facilitate ODAEH, the context in which such a request is made at least in part contributes to granting or refusing such request. For example, now the intensive care units are “flooded” with COVID patients, and hospitals can barely facilitate emergency and urgent surgeries, ODAEH is currently not an option.

Ethical considerations for every team member have to be evaluated as well. Every potential team member should be one hundred percent supportive of this procedure, and be certain they do not have any moral, religious or cultural objections to the procedure. Only the people that where fully committed participated in honouring the last wishes of the patient. Before and after the procedure several debriefing moments were scheduled with all participants.

## Conclusion

Organ donation after euthanasia is increasingly requested and performed in the Netherlands, Belgium and Canada. Giving a patient the possibility to be sedated at home prior to transport to the hospital, and performing euthanasia directly next to the operating room, all at the patient’s request, maximally contributes to the patient’s autonomy.

From the patient’s perspective, falling asleep in the intimacy of his own home and with his loved ones present appears to be a good alternative to the medicalisation of organ donation after euthanasia in the hospital.

Even though this expansion of the combined euthanasia and organ donation procedure adds medical, legal and ethical challenges to an already complex situation, it provides the patient the possibility to ‘fall asleep’ at home and to help other patients who are on the transplant waiting list. The dialogue on the acceptability and feasibility of ODAEH is ongoing. Since the procedure is so far uncommon in occurrence, it is not yet incorporated in the most recent version of the Dutch national guideline on organ donation after euthanasia.

Normalizing ODAEH could make it more attractive to patients who want to donate after euthanasia but do not want to die in hospital, thus providing more organs for transplantation. Given the prospective benefits, the marginal and relative extra costs of implementing this procedure are likely to be justified.

### Note

The patient and his relatives were strongly in favour and supportive of publication of this case to contribute to public awareness of organ donation after euthanasia. Therefore the patient’s wife is one of the authors of this article (AK)

## Data Availability

Not applicable.

## Abbreviations

DCD: Donation after circulatory death
ODAEH: Organ donation after euthanasia starting at home
